# Biometrics and Biosecurity 2014

**DOI:** 10.1155/2015/812697

**Published:** 2015-07-21

**Authors:** Tai-hoon Kim, Sabah Mohammed, Wai-Chi Fang, Carlos Ramos

**Affiliations:** ^1^University of Tasmania, Centenary Building, Room 350, Private Bag 87, Hobart, TAS 7001, Australia; ^2^Lakehead University, 955 Oliver Road, Thunder Bay, ON, Canada P7B 5E1; ^3^National Chiao Tung University, 1001 University Road, Hsinchu 300, Taiwan; ^4^ISEP/IPP, Rua Dr. António Bernardino de Almeida 431, 4200-072 Porto, Portugal

This issue contains several articles that come from various countries, among which we mention German, Brazil, Korea, and Japan.

Biometrics and Biosecurity focused on the various aspects of advances in biometrics and biosecurity. This special issue will provide a chance for academic and industry professionals to discuss recent progress, problems, and solutions in the area of biometrics and its application, biosecurity measures, and biosafety protocols, including development, implementation, strategies, and policies.

As a novel approach to perform user authentication, authors proposed a multimodal biometric system that uses faces and gestures obtained from a single vision sensor in the paper “A Multimodal User Authentication System Using Faces and Gestures.” Unlike typical multimodal biometric systems using physical information, the proposed system utilized gesture video signals combined with facial images.

The purpose of the paper “Quantification of Hepatorenal Index for Computer-Aided Fatty Liver Classification with Self-Organizing Map and Fuzzy Stretching from Ultrasonography” was to show that HRI is an important and informative diagnostic attribute in multiclass fatty liver classification because of such quantification. This encouraged authors to develop reliable automatic diagnostic software if it is combined with other sets of useful textual or statistical features and other powerful machine learning algorithms in the future.

The aim of the paper “Towards a Food Safety Knowledge Base Applicable in Crisis Situations and Beyond” was to verify that a framework established for efficient and transparent conduction of exposure assessments in the food sector could also be applied in case of bio- and agroterroristic crisis situations. For this, data and models on tenacity of highly pathogenic agents were collected and applied in sample scenarios together with knowledge on relevant food production processes.

In “Establishing Standards for Studying Renal Function in Mice through Measurements of Body Size-Adjusted Creatinine and Urea Levels” authors showed that creatinine clearance measurements should be adjusted according to the body surface area, which was calculated based on the weight and length of the animal. Authors' findings will facilitate standardization and optimization of methodology as well as understanding of renal and other biochemical data obtained from mice.

In the paper “Biometrics Analysis and Evaluation on Korean* Makgeolli* using Brainwaves and Taste Biological Sensor System” authors conducted sensory evaluation, whereas a maximum of nine points were accumulated by purchasing eight types of rice wine. The contribution of this paper was to overcome the disadvantages of the sensory evaluation with the usage of the suggested taste biological sensor system.

In the paper “A Multi-layer secure Biomedical Data Management System for Remotely Managing a Very Large Number of Diverse Personal Healthcare Devices” a multilayered remote PHD management system for a very large number of PHDs was proposed. Some experiments, including the stress test, were carried out to show that the system proposed in this paper performed very well even when a very large number of PHDs were 30 used.

